# Supplementary material for: Identification of New Candidate Genes Related to Semen Traits in Duroc Pigs through Weighted Single-Step GWAS
Source: Animals (Basel). 2023 Jan 20;13(3):365. doi: 10.3390/ani13030365 (PMC9913471; doi:10.3390/ani13030365)
Supplement: Supplementary file 1 [file animals-13-00365-s001.zip › animals-2105881-supplementary.pdf]

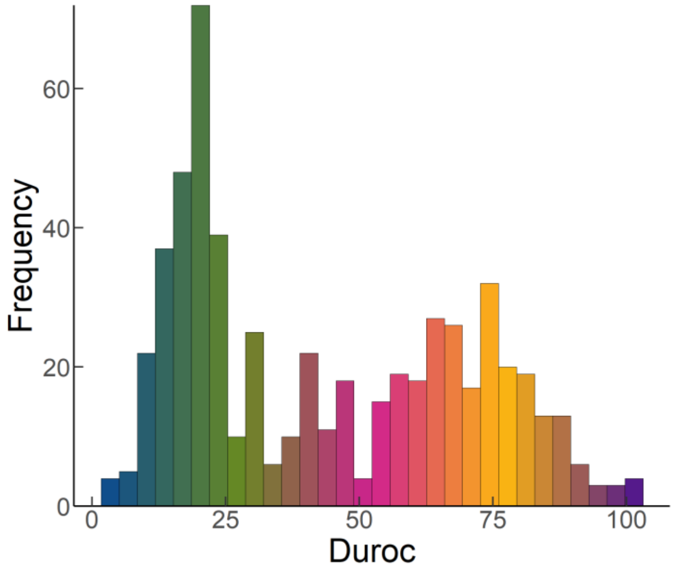

Figure S1. Distribution histogram with ejaculation times of Duroc pigs.

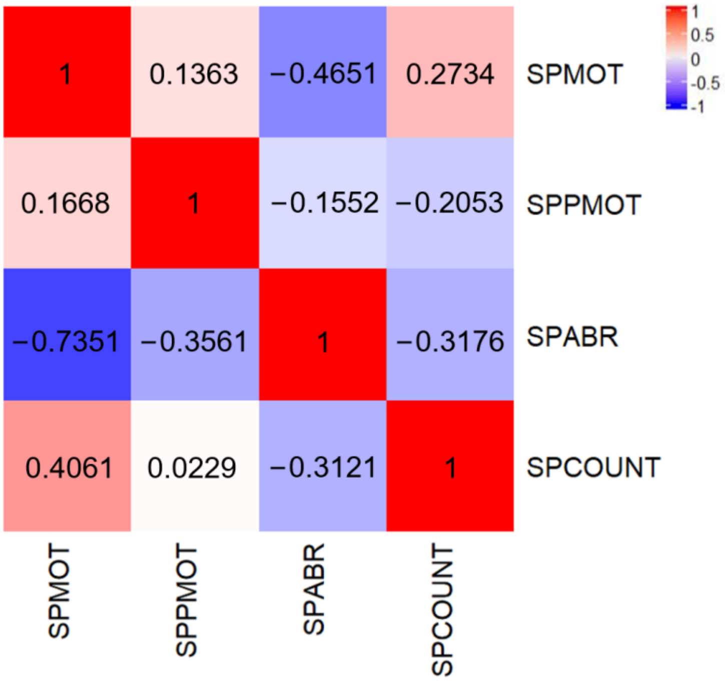

Figure S2. Genetic and phenotypic correlation for semen traits of Duroc pigs. The lower triangle is genetic correlation and the upper triangle is phenotypic correlation.

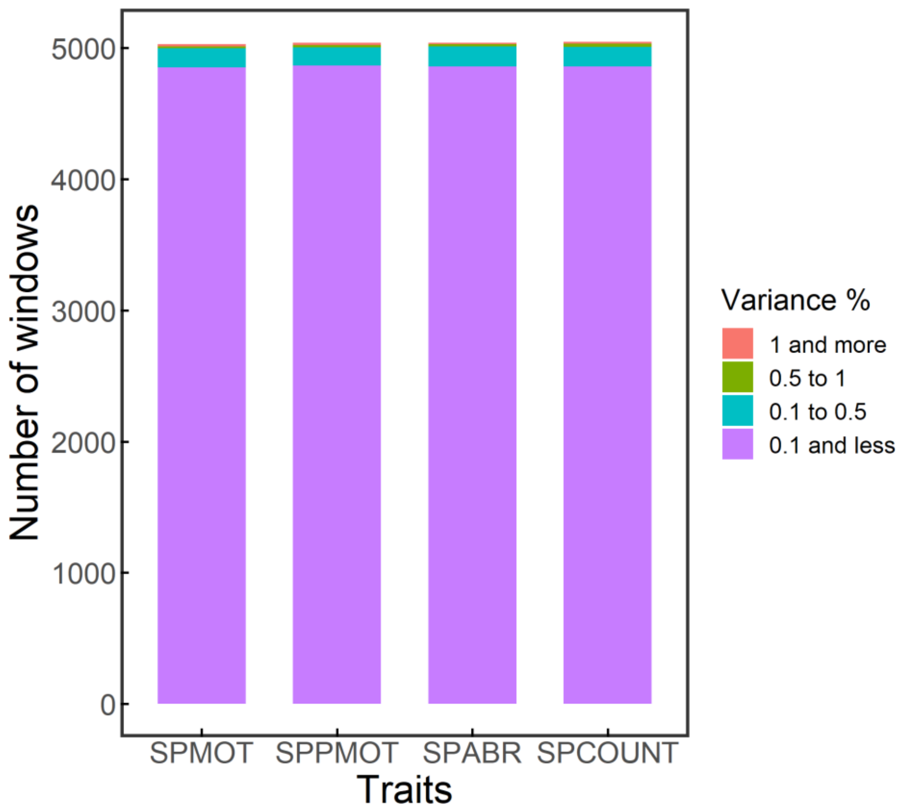

Figure S3. Distribution of the four classes in four semen traits of Duroc pigs (the 0.4 Mb regions explained genetic variance: >1%, 0.5%–1%, 0.1%–0.5%, and <0.1%.).

Table S1. All candidate genes for semen traits of Duroc pigs.

Trait [a]	Count	Candidate Gene
SPMOT	59	*CNOT4, STRA8, WDR91, TMEM140, CALD1, BPGM, AKR1B1, ADORA2B, TTC19, ZSWIM7, PMP22, TEKT3, TRIM16, ZNF624, ZNF287, LRRC75A, TRPV2, UBB, PIGL, NPAS4, SLC29A2, B4GAT1, BRMS1, TMEM151A, YIF1A, RAB1B, PACS1, SF3B2, GAL3ST3, CATSPER1, CST6, EIF1AD, SART1, TSGA10IP, C11orf68, DRAP1, FOSL1, FIBP, CTSW, EFEMP2, MUS81, CFL1, SNX32, OVOL1, AP5B1, KAT5, RNASEH2C, RELA, SIPA1, PCNX3, MAP3K11, KCNK7, EHBP1L1, FAM89B, ZNRD2, LTBP3, SCYL1, CD248, CCDC85B*
SPPMOT	8	*PTBP2, CNOT4, STRA8, WDR91, TMEM140, CALD1, BPGM, AKR1B1*
SPABR	36	*ADORA2B, TTC19, ZSWIM7, PMP22, TEKT3, TRIM16, ZNF624, ZNF287, LRRC75A, TRPV2, UBB, PIGL, NPC2, ISCA2, LTBP2, AREL1, FCF1, YLPM1, PROX2, DLST, RPS6KL1, EIF2B2, MLH3, ZC2HC1C, NEK9, TMED10, FOS, ssc-mir-423, EFCAB5, BHLHA9, NSRP1, SLC6A4, BLMH, TMIGD1, CPD, GOSR1*
SPCOUNT	28	*TSPAN18, CD82, ALX4, ACCS, EXT2, ACCSL, CCDC70, NEK3, NEK5, ALG11, TMEM272, WDFY2, INTS6, SERPINE3, FAM124A, LRP4, CKAP5, F2, ZNF408, ARHGAP1, ATG13, HARBI1, AMBRA1, MDK, CHRM4, DGKZ, CREB3L1, PHF21A*

[a] SPMOT: sperm motility; SPPMOT: sperm progressive motility; SPABR: sperm abnormality rate; SPCOUNT: total sperm count. Within each trait, genomic regions were decreasingly sorted based on the proportion of genetic variance explained.

Table S2. GO terms where the candidate genes were significantly ($p < 0.05$) enriched.

Term [a]	Count	*p*-Value	Candidate Genes
GO:0022414—reproductive process	3	0.000192	*CATSPER1UBB, STRA8*
GO:0000003—reproduction	3	0.000198	*CATSPER1, UBB, STRA8*
GO:0051321—meiotic cell cycle	2	0.000347	*UBB, STRA8*

GO:0007018—microtubule-based movement	2	0.000789	*CATSPER1, UBB*

[a] GO, Gene Ontology.
